# Impact of the COVID-19 Pandemic on the UNAIDS Six 95% HIV Control Targets

**DOI:** 10.3389/fmed.2022.818054

**Published:** 2022-02-18

**Authors:** Tiande Jiang, Cuie Liu, Jing Zhang, Xiaojie Huang, Junjie Xu

**Affiliations:** ^1^Department of Dermatology, Beijing Youan Hospital, Capital Medical University, Beijing, China; ^2^NHC Key Laboratory of AIDS Immunology (China Medical University), National Clinical Research Center for Laboratory Medicine, The First Affiliated Hospital of China Medical University, Shenyang, China; ^3^Center for Infectious Disease, Beijing Youan Hospital, Capital Medical University, Beijing, China; ^4^Peking University Shenzhen Hospital, Clinical Research Academy, Peking University, Shenzhen, China

**Keywords:** COVID-19, HIV, six 95% HIV control targets, policy, HIV key population

The Joint United Nations Program on AIDS/HIV (UNAIDS) 90-90-90 human immunodeficiency virus (HIV) 2020 control targets have been replaced by the ambitious six “95” targets to end the HIV disease pandemic by 2025 (1). The 95-95-95 HIV control targets are the core of the six 95% HIV control targets and are updated goals compared to the 90-90-90 goal at meeting the needs of people living with HIV (PLWH). In contrast, the coronavirus disease (COVID-19) pandemic led to a “build back better” approach to setting the 2025 HIV control targets (2).

The first three “95” targets have been heavily impacted by the COVID-19 pandemic. These targets are as follows: that 95% of people living with HIV would know their HIV status, that 95% of PLHIV who know their HIV status would be on antiretroviral therapy (ART), and that 95% of people on ART to have suppressed viral loads (1). Among the first three targets, the first “95” target is the most affected since the start of the COVID-19 pandemic (3), especially in the low and middle-income countries and regions. The proportion of HIV tests decreased, ranging from 47.6 to 80% in April 2020 in South Africa (4) and Peru (5). Between April and June 2020 alone, the COVID-19 has led to about a 30% reduction in the use of HIV testing services in communities and health facilities in Uganda (6). Compared to the pre-COVID-19 period, there was about 20% reduction of HIV tests in Italy (7). In addition, since more than half of HIV clinics had stopped in-person visits and blood testing and focused on ART drug distribution to patients diagnosed before the pandemic (8), the second “95%” target of ART might be the least impacted. A study of 65 South African primary care clinics found that the median number of ART initiations per week decreased from 571 before the lockdown to 375 per week (4). During COVID-19, ART initiation has decreased by 31% in Uganda within 2 months. Currently, there is little data on the third “95%” target. This is due to the testing of HIV viral load and CD4+ T cell counts being severely impacted because of the manufacturers of viral load testing platforms having to instead develop the molecular diagnostic capability for SARS-CoV-2 using the same equipment. Consequently, many laboratory staff members were shifted from molecular testing for HIV to testing for SARS-CoV-2. Between December 2019 and June 2020, HIV viral load testing coverage has decreased from 96 to 85%, and CD4 access decreased from 31 to 22% in Uganda (6). Meanwhile, many PLWH cannot afford or access medications because of lost income and health insurance during the COVID-19 pandemic (6). Also, due to transportation challenges, the delivery of orders for some international shipments of antiretroviral drugs has been delayed (9). Mathematical models predict that if 50% of the PLWH being treated were to experience a 6-month interruption of ART, the number of HIV-related deaths would increase by 1.63 times the original rate (10). A disruption as small as 20% could cause an additional 110,000 deaths (10). By extension, a six-month complete disruption in ART could cause more than 500,000 additional deaths in sub-Saharan Africa from 2020 to 2021, bringing the region back to 2008 AIDS mortality levels. Centralizing patients and vulnerable populations while decentralizing HIV care is anticipated for HIV/AIDS control in the COVID-19 era (11) (Table 1).

**Table 1:** The impact of COVID-19 on the six “95” HIV control targets and the opportunities for the future.

**Targets**	**The COVID-19 impact**	**Strength of the impact**	**Opportunities for the future**
95% of PLHIV know their HIV status.	• Reduction in number of HIV-tests.	• The number of HIV-tests decreased ranging from 47.6 to 80% in April 2020 in South Africa and Peru, 30% in Uganda, and 20% in Italy.	• Increasing the availability of HIV self-testing and rapid test screening in non-hospital settings including community, instead of in-person visits.
95% of PLHIV who know their status initiate treatment.	• Decreased number of ART initiations.	• The median number of ART initiations per week decreased from 571 before the lockdown to 375 per week after the lockdown in 65 South African primary care clinics. • The initiation of ART has decreased by 31% between April and June 2020 alone in Uganda.	• The near-real-time daily data on COVID-19 cases and mortality reporting addresses surveillance in managing a major pandemic, which can be used in HIV cases management.
95% on treatment are virally suppressed.	• Patients dropped out of treatment. • Decreased ability to monitor treatment outcomes with viral load testing. • Drug shortages.	• A six-month complete disruption in ART could cause more than 500,000 additional deaths in sub-Saharan Africa during 2020–2021, bringing the region back to 2008 AIDS mortality levels. Even a 20% disruption could cause an additional 110,000 deaths. • Manufacturers of viral load testing platforms developed molecular diagnostic capability for SARS-CoV-2 using the same equipment used for HIV viral load testing. Many laboratory staff members were shifted from molecular testing for HIV to testing for SARS-CoV-2. HIV Viral load testing coverage has decreased from 96 to 85%, and CD4 access decreased from 31 to 22% in Uganda between December 2019 and June 2020.	• Centralizing patients and decentralizing HIV care, including multi-month dispensing and expanding collection of dried blood spot specimens, directly delivering ART to communities, and integrating HIV viral load testing with ART distribution.
95% coverage of services for eliminating vertical transmission.	• The COVID-19 pandemic further decreases the proportion of women tested for HIV at the first antenatal clinic visit and their use of ART.	• Among the 15 countries reporting treatment among pregnant women living with HIV, all but five have recovered to the February numbers of women receiving treatment (except Botswana, South Africa, Sierra Leone, Togo, and Guatemala).	• Maintaining efforts as pre-COVID-19 period.
95% of women access HIV and sexual and reproductive health services	• The COVID-19-related mitigation strategies drove adolescent girls away from school, which could reduce their vulnerability to HIV infection.	• All countries except Mozambique and Jamaica experienced declines in women tested for HIV at their first antenatal clinic visit in April compared to January. By June or July, 14 of the 17 countries were back to the February level of testing (all except Indonesia, Botswana, and Sierra Leone).	• Laws should safeguard women's rights and interests and guarantee access to medical supplies.
95% use combination prevention	• Impeded access to prevention measures, including STIs screening and access to HIV PrEP, etc.	• An 85% reduction in HIV/gonorrhea/chlamydia tests was reported in the Boston area's first quarter of the year. • A report from Lombardy, Italy, showed a rise in acute bacterial STIs during the period of lockdown (5). • A report from Melbourne showed that 25% of PrEP users stopped taking PrEP during the COVID-19 outbreak.	• Availability of a combination of self-testing kits for STIs and HIV testing. • Transferring links for HIV PrEP.

While non-essential outpatient services are being reduced or suspended and COVID-19 is diverting attention from PLWH and vulnerable populations (8), it is essential to reaffirm the new HIV control targets to strengthen the vulnerable subpopulation who were most impacted by COVID-19. The additional new three “95%” targets are newly added as follows: 95% of women of reproductive age who have their HIV and sexual and reproductive health service needs met, 95% of pregnant and breastfeeding women living with HIV to achieve viral suppression, and 95% of people at risk of HIV infection to use appropriate, prioritized, person-centered, and effective combination prevention options by 2025 (1). The impact on services for the prevention of vertical transmission of HIV (from mother to child) is mixed. By April 2020, countries generally saw a decline in the number of women tested for HIV at their first antenatal clinic visit, but by June 2020, that decline had been reversed (12). Structural challenges to centering adolescent girls and young women in the HIV response, such as poverty, gender inequality, and gender-based violence, existed before the COVID-19 pandemic (13). The COVID-19 pandemic further decreases the proportion of women tested for HIV at their first antenatal clinic visit and their use of ART (14). Evidence shows that keeping adolescent girls in school reduces their vulnerability to HIV infection. The COVID-19-related mitigation strategies further increase the risk of pregnancy, child labor, and violence among women (15). There was evidence that the usage of combination prevention has been impacted during COVID-19, including sexually transmitted infection screening and access to pre-exposure prophylaxis (PrEP). An 85% reduction in HIV/gonorrhea/chlamydia tests was reported in the first quarter of the year 2020 in the Boston area. The same reduction was reported in Lombardy, Italy (7). A report from Melbourne showed that 25% of PrEP users stopped taking PrEP during the COVID-19 outbreak (16) (Table 1).

The COVID-19 pandemic creates both pressure and opportunities to attain the six “95” targets (Table 1). We learned and mastered new tools from the COVID-19 pandemic. For the first three 95% targets of HIV testing, ART initiation, and ART adherence in particular, the opportunity lies in decentralizing HIV care and centralizing patients and vulnerable populations, including expanding non-facility-based telemedicine and delivery modes, distribution of HIV/STIs self-testing and collection of dried blood specimens, multi-month dispensing of ART (17), and strengthening the role of community-based organizations (CBOs). In cooperation with these, the near-real-time daily data on COVID-19 cases and mortality reporting addresses surveillance in managing a major pandemic. Promptly monitoring HIV-related data helps to formulate plans to attain the 2025 goals. On the other hand, for the latest three 95% targets, laws should safeguard the rights and interests of women in addition to guaranteeing access to medical supplies. Moreover, virtual communication platforms, such as providing anti-partner violence, mental health services, and promoting knowledge and awareness of sexual health and PrEP, that aim to help vulnerable populations are innovative and necessary.

## Author Contributions

TJ, CL, XH, and JX were involved in designing and designing of the study. TJ, JZ, XH, and JX drafted the report. All authors reviewed and approved the final manuscript.

## Funding

This work was supported by the National Science and Technology Major Project of China during the 13th Five-Year Plan Period (2017ZX10201101), the Beijing Excellent Talent Plan (2018000021223ZK04), the Beijing Talent Project in the New Millennium (2020A35), the National Natural Science Foundation of China (81872674), and the National Science and Technology Major Project (2018ZX10101001-001-003).

## Conflict of Interest

The authors declare that the research was conducted in the absence of any commercial or financial relationships that could be construed as a potential conflict of interest.

## Publisher's Note

All claims expressed in this article are solely those of the authors and do not necessarily represent those of their affiliated organizations, or those of the publisher, the editors and the reviewers. Any product that may be evaluated in this article, or claim that may be made by its manufacturer, is not guaranteed or endorsed by the publisher.
